# A Superhydrophobicity–Slipperiness Switchable Surface with Magneto- and Thermo-responsive Wires for Repelling Complex Droplets

**DOI:** 10.1021/acs.langmuir.3c03556

**Published:** 2024-01-22

**Authors:** Chuanqi Wei, Oleg Gendelman, Youhua Jiang

**Affiliations:** † Department of Mechanical Engineering (Robotics), Guangdong Technion—Israel Institute of Technology, Shantou, Guangdong 515063, China; ‡ Faculty of Mechanical Engineering, Technion—Israel Institute of Technology, Haifa 3200003, Israel; § Guangdong Provincial Key Laboratory of Materials and Technologies for Energy Conversion, Guangdong Technion—Israel Institute of Technology, Shantou, Guangdong 515063, China

## Abstract

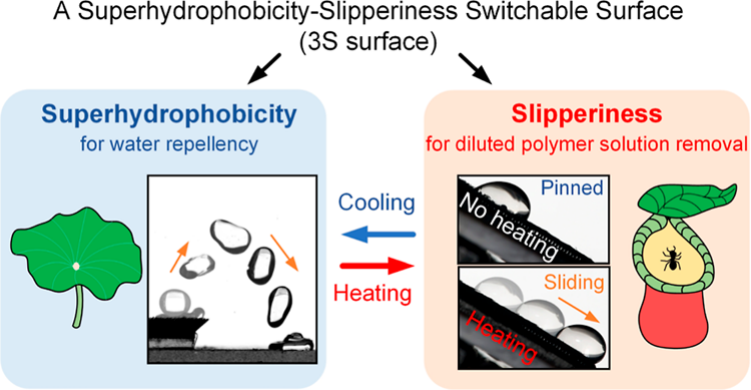

The inefficacy of repelling water droplets laden with macromolecules (complex droplets or diluted polymer solution) is a long-standing shortcoming of superhydrophobic surfaces, which severely limits their reliability in practical applications. Here, we design a surface termed the superhydrophobicity–slipperiness switchable surface (3S surface), which demonstrates superhydrophobicity at room temperature and slipperiness when heated. The 3S surface is composed of magneto-responsive wires coated with superhydrophobic nanoparticles and impregnated with thermoresponsive paraffin, exhibiting lotus leaf-inspired passive water repellency and respiratory cilia-inspired active water repellency at room temperature. When heated, the impregnated paraffin melts and forms a lubricant layer atop the surface structures, exhibiting the pitcher-plant-inspired removal of complex droplets that remain pinned on conventional superhydrophobic surfaces. The counterintuitive integration of superhydrophobicity (a liquid–solid–gas composite system) and slipperiness (a liquid–lubricant–gas system) into a surface and the on-demand switch between them are not only important to the applicability of self-cleaning surfaces to real-world environments, where complex liquids are inevitable, but also provide insights into various interface-related applications.

## Introduction

**Figure 1:**
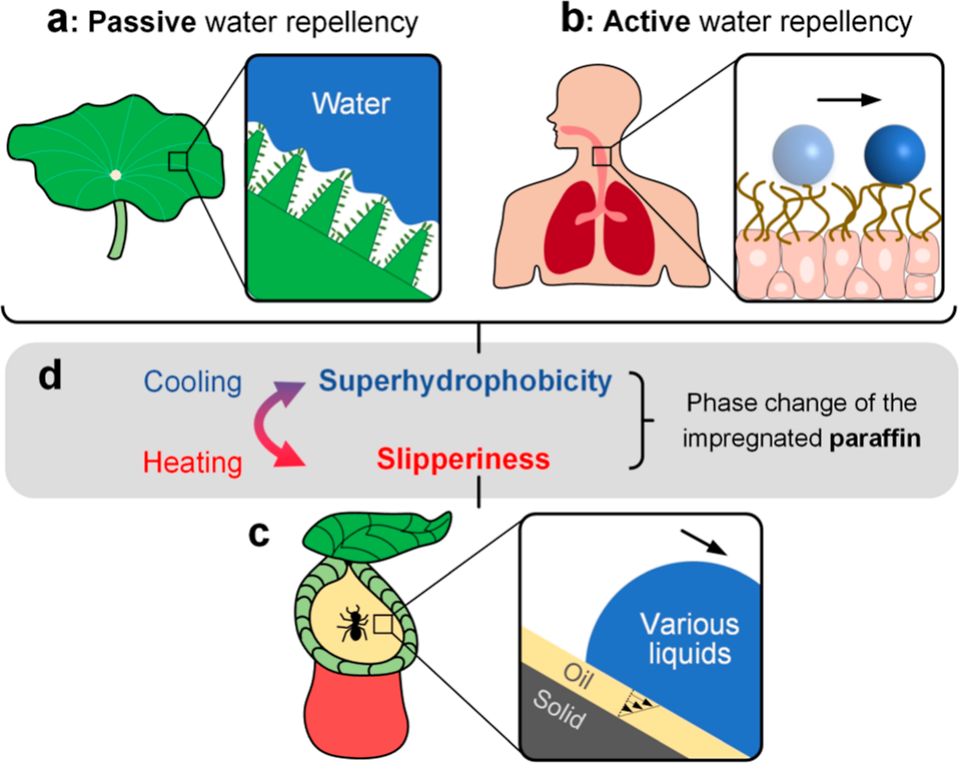
Schematical introduction of the superhydrophobicity–slipperiness switchable surface (3S surface), which mimics (a) lotus leaves for passive water repellency, (b) respiratory cilia for active water repellency, and (c) pitch plants for slipperiness that removes various liquids. The former two represent surface superhydrophobicity and the latter indicates surface slipperiness, and (d) switch between them is allowed by thermoresponsive impregnated paraffin.

**Figure 2:**
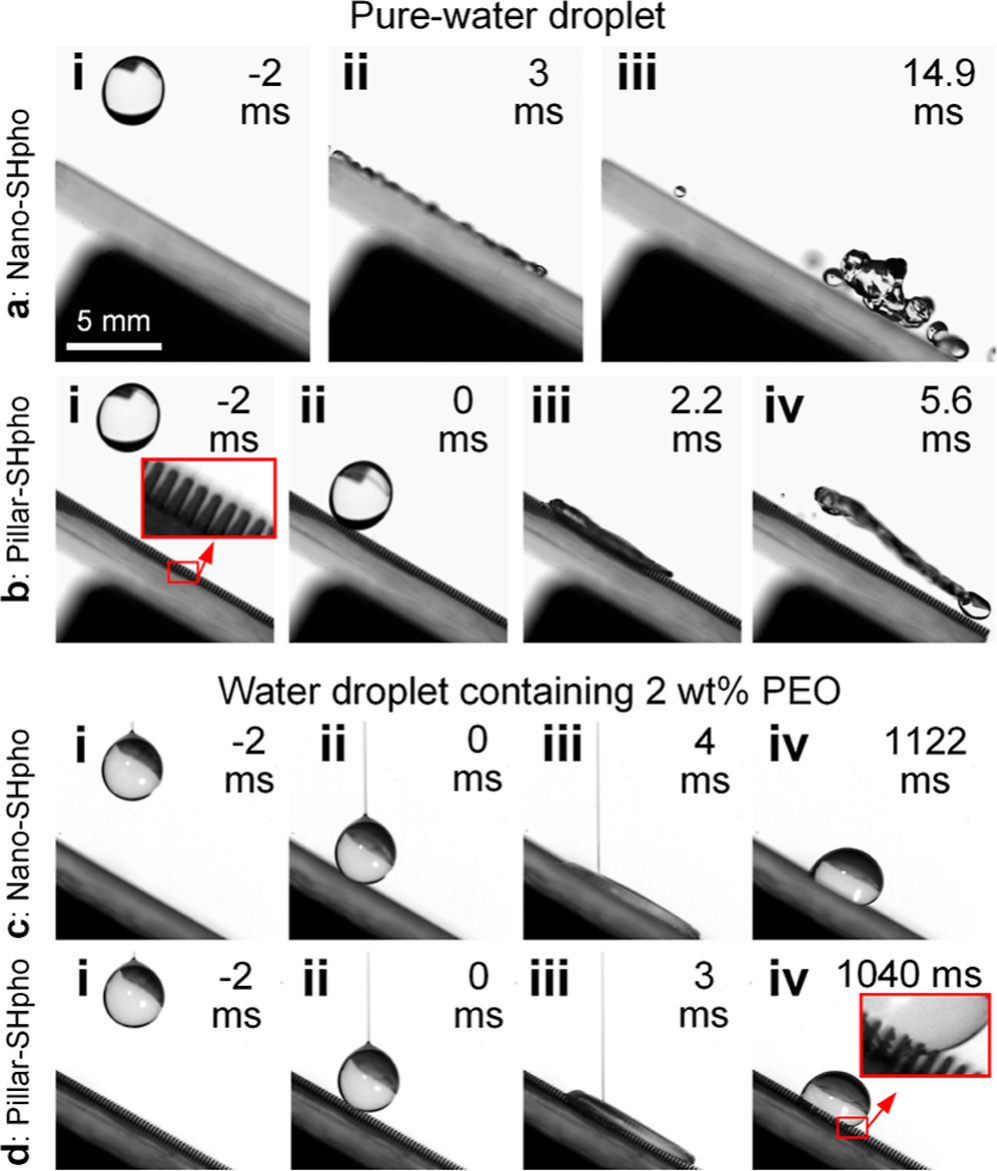
Demonstration of excellent water repellency of a (a) superhydrophobic surface with nanostructures (Nano-SHpho) and (b) superhydrophobic surface with millimetric pillars and nanostructures (Pillar-SHpho), where the latter exhibits droplet pancake-bouncing. (c,d) Failure of those surfaces (Nano-SHpho and Pillar-SHpho) in repelling water droplets containing 2 wt % PEO. The moment at the initial droplet–substrate contact is *t* = 0 ms. The “tail” on the droplets in (c,d) reflects the liquid viscoelasticity. The inset is a zoomed-in image to show the pillar geometry and the pinned droplet boundary. Droplet volumes are 20 μL and impacting speeds are 2.1 m/s.

Over the past decades, superhydrophobic surfaces have gained extensive attention from researchers in various fields, ranging from fundamental physics and mechanics, energy systems, environmental science, to biotechnology,^1−10^ because of their intriguing water-repellent characteristics. The explorations on superhydrophobic surfaces have advanced significantly from lotus leaf-inspired surfaces that passively repel water and some low-surface tension liquids^11−13^ (Figure 1a) to respiratory cilia-inspired surfaces that remove impurities in an active manner (Figure 1b).^14−18^ However, it is a longstanding challenge of superhydrophobic surfaces that they typically cannot repel complex droplets,^19−21^ e.g., water droplets containing macromolecules (diluted polymer solution), because of complicated reasons relating to rheological characteristics within the bulk droplet (non-Newtonian fluid) and near the contact line and macromolecule–substrate interactions, etc.^22−26^ Specifically, as shown in Figure 2a, a ∼20 μL water droplet with an impacting speed of 2.1 m/s completely rebounded from a superhydrophobic surface coated with hydrophobic SiO_2_ nanoparticles (denoted as Nano-SHpho surface). In addition, the pancake-bouncing phenomena,^1^ which manifest the superior water repellency of a surface, were observed on a superhydrophobic surface decorated with millimetric pillars and SiO_2_ nanoparticles (denoted as Pillar-SHpho), and the contact time was as low as 5.6 ms (Figure 2b). Nevertheless, both surfaces failed in repelling PEO-laden droplets, i.e., water droplets containing 2 wt % poly(ethylene oxide), as shown in Figure 2c,d. This challenge ought to be overcome because complex droplets are inevitable in real-world applications, ranging from our daily lives, biomedical devices, chemical plants, to the petroleum industry.

Lubricant-impregnated surfaces allow the removal of complex droplets (in fact, various liquids) because of a lubricant layer in between the complex droplet and the surface structures (a functionality denoted as slipperiness),^27−30^ where the solid–liquid direct contact is minimized and the droplet motion is controlled mainly by lubricant’s shear stress (Figure 1c). However, lubricant-impregnated surfaces require a driving force to remove droplets (typically gravitational force with a substrate inclination or Laplace pressure force with a substrate curvature gradient) and cannot allow droplet rebound for self-cleaning purposes, like the case occurring on superhydrophobic surfaces because of the high droplet–lubricant normal adhesion. Therefore, a question arises: is it possible to design a surface that exhibits either superhydrophobicity or slipperiness when needed?

Nowadays, thanks to stimuli-responsive surfaces, the switch between superhydrophobicity and slipperiness has been achieved under certain conditions.^31−33^ For example, iron-laden and lubricant-impregnated pillars allowed the rebound of impacting water droplets (superhydrophobicity) with very small impacting speeds (<0.4 m/s) because the Cassie–Baxter wetting state provided by the discrete pillar arrays sustained.^31^ On the other hand, the iron-laden pillars were deflected under a magnetic field so that the impacting droplet contacted the continuous lubricant film without rebound (slipperiness). Nevertheless, the applicability of such surfaces to harsh operating conditions, e.g., droplets with high impacting speeds (large Weber numbers), droplets laden with macromolecules (diluted polymer solution), and horizontally placed surfaces that cannot utilize gravity as the driving force for impurity removal, remains questionable.

**Figure 3:**
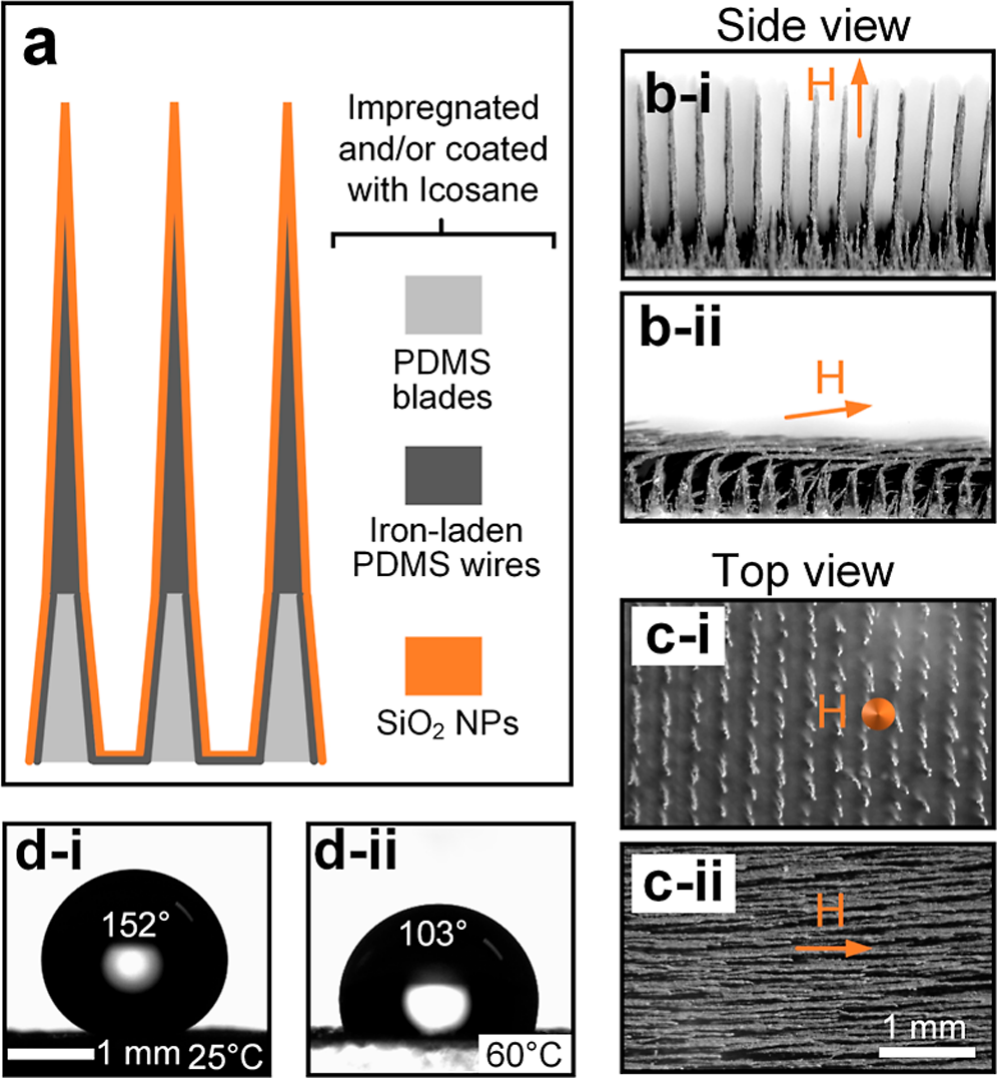
(a) Configuration of the 3S surface, including PDMS blade arrays, iron-laden PDMS wires, SiO_2_ nanoparticle coatings, and the impregnated paraffin (icosane), which should also appear within the PDMS matrix but is not drawn. (b) Side and (c) top views of the 3S surface with a magnetic field (0.45 T) applied (i) perpendicular and (ii) parallel to the 3S surface substrate. The directions of the magnetic field lines (*H*) are indicated by the arrows. (d) Droplet shape profiles on a (i) cold and (ii) heated flat PDMS surface coated with hydrophobic SiO_2_ nanoparticles and impregnated with paraffin. The transparent (bright) substrate in (ii) indicates the melting of the impregnated paraffin.

Aiming at addressing the above concerns and limitations, here, we introduce a surface referred to as the superhydrophobicity–slipperiness switchable surface (3S surface), which demonstrates superhydrophobicity at room temperature and slipperiness when heated; the superhydrophobicity-to-slipperiness transition is achieved by using a thermoresponsive paraffin (Figure 1d). The 3S surface is composed of four key components (Figure 3a): polydimethylsiloxane (PDMS) blade arrays for structure alignment, iron-laden PDMS wires for magneto-responsiveness, hydrophobic SiO_2_ nanoparticles for superhydrophobicity, and impregnated paraffin within the PDMS matrix for slipperiness. The 3S surface exhibits lotus leaf-inspired passive water repellency and respiratory cilia-inspired active water repellency at room temperature. When encountering complex droplets that are sticky to superhydrophobic surfaces, the 3S surface is heated and the impregnated paraffin melts, forming a lubricant layer atop the surface structures that allows the pitcher plant-inspired removal of complex droplets.^34−36^

## Experiments and Methods

### Fabrication of Well-Ordered Magneto-Responsive Structures

The detailed procedures of surface fabrication can be found in our prior work in ref (17). Specifically, groove arrays were drilled on polished aluminum plates using a laser driller (HS50II, Han’s Group, China). Then, replication of the groove arrays was conducted using PDMS solution (Sylgard 184, Dow Corning, USA) with a base-to-curing agent ratio of 10:1, resulting in blade arrays with a height of 0.6 mm, center-to-center spacing of 0.3 mm, and width of 0.1 mm (see Figure S1a–d in Supporting Information).

Iron particles with average diameters of around 5 μm (Macklin, China), a PDMS solution with a base-to-curing agent ratio of 10:1, and toluene (Xilong Scientific, China) were mixed at a weight ratio of 3:2:6. Then, iron-laden PDMS aerosols were atomized and sprayed toward the PDMS blade arrays which were placed atop a neodymium permanent magnet with a strength of 0.45 T (Shenzhen Minci Technology, China). Under this magnetic field, aerosols aggregated following the magnetic field lines with a preferred aggregation location along the blade tips, resulting in iron-laden PDMS wires formed atop blade tips. After heat-curing, wires with a length *L*_w_ of around 1.4 mm (wire and blade total length of 2.0 mm), diameter *d*_w_ of around 70 μm, and length-to-diameter aspect ratio of 30 were formed (Figure S1e,f).

### Superhydrophobization of Surface Structures

Hydrophobic SiO_2_ nanoparticles with diameters ranging from 7 to 40 nm (3A Materials, China), 1*H*,1*H*,2*H*,2*H*-perfluorodecyltriethoxysilane, (3-aminopropyl)triethoxysilane (Aladdin, China), and ethanol (Xilong Scientific, China) were mixed at a weight ratio of 1:1:1:50 and were spray-coated on the surface (Figure S1g), followed by heating at 80 °C in an oven for 1 h.

### Impregnation of Structures with Paraffin

The as-prepared surface was first weighted by a microbalance and then immersed in a tank filled with melted paraffin (icosane, C_20_H_42_, Aladdin, China) at 80 °C for 1 h (Figure S1h).^34−36^ The icosane has a density of around 789 kg/m^3^, melting point at around 36 °C, surface tension of 0.026 N/m at 60 °C, and viscosity μ of 2.8 mPa·s at 60 °C. After retrieving the surface from the tank, the excess paraffin on the surface was removed by placing the surface upside-down over a dustless wipe for a period ranging from 5 s to 2 h (denoted as the paraffin-removing time) while the surface remained heated, followed by weighting the surface again. The impregnated paraffin following the above procedure weighed from 10 to 30 wt % of the processed surface, which decreases with the increase in the paraffin-removing time (Figure S2). Unless specifically noted, experiments were conducted using 3S surfaces with 10 wt % paraffin.

### Characterization of Surface Wettability

The surface morphology and its deflection in response to a magnetic field was visualized by a digital camera (D5600, Nikon, Japan) attached with a microlens (Navitar, USA). The wire inclination angle β was controlled by relocating the magnetic field, where wires perpendicular to the substrate is β ≈ 0° [Figure 3b(i)] and those in parallel to the substrate is β ≈ 90° [Figure 3b(ii)]. To characterize the surface wettability rendered by hydrophobic SiO_2_ nanoparticles and the impregnated paraffin, an identical surface treatment was conducted on a flat PDMS substrate without blade arrays or wires. At room temperature (25 ± 2 °C), where the paraffin is solidified, the treated flat PDMS mainly demonstrated superhydrophobicity [Figure 3d(i)] with the advancing (θ_a_) and receding (θ_r_) contact angle of deionized water droplets (liquid–gas interfacial tension γ_LG_ of 0.072 N/m) measured to be 155 ± 2 and 145 ± 2°, respectively. When this surface was heated, the paraffin melted, and therefore the surface exhibited slipperiness [Figure 3d(ii)] with θ_a_ and θ_r_ of 105 ± 2 and 100 ± 2°, respectively. The contact angles of water droplets heavily depend on wire configuration (e.g., wire deflection angle β), and hence the contact angles on 3S surfaces were not reported.

### Impact and Coalescence of Water Droplets

A water droplet (∼10 μL) was released from a micropump (PHD ULTRA, Harvard Apparatus, USA) and allowed to impact onto 3S surfaces with various wire configurations. The droplet impact speeds, *v*, were adjusted by droplet release heights. In addition, two water droplets (∼10 μL) were placed next to each other on the 3S surface with inclined wires (inclination angle β ≈ 45°) and were allowed to merge. The droplet dynamics were captured by a high-speed camera (Nova S16, Photron, Japan) at a speed of 10,000 frames per second (fps), and *v* were measured from the videos.

### Active Removal of Impurities

Regarding the impurity removal, a magnet (0.45 T) was placed on a linear stage (QRXQ-700, Ruixin Tech, China). A glass slide with a fixed end was placed atop the linear stage and the 3S surface was placed on this glass slide.^17^ This configuration allowed the magnet to slide underneath the 3S surface. Water droplets of ∼10 μL in volume and polyoxymethylene (POM) beads with diameters of 2.5 mm were used for demonstration.

### Impact and Removal of Complex Liquids

A droplet (∼20 μL) containing 2 wt % high-molecular-weight (molecular weight of 4 × 10^6^ g/mol) poly(ethylene oxide) (PEO, Aladdin, China), i.e., diluted polymer solution, was allowed to impact onto a 30°-titled 3S surface with completely deflected wires (β ≈ 90°). After the complex droplet completely pinned, a voltage of 9 V was applied to a thermoelectric heater (Xinxin Century Industry, China) placed underneath the 3S surface, resulting in a temperature of ∼60 °C at the top of the 3S surface measured by a thermocouple (type T, Kaipusen, China). The droplet removal was then recorded with a digital camera (D5600, Nikon, Japan) at 60 fps. The displacement of the droplet center with respect to time was measured by software called Tracker.

The viscoelastic properties of such complex (non-Newtonian) fluids were measured by a rheometer (MCR 102e, Anton Paar, Austria) and detailed in Figure S3,^37^ and γ_LG_ was measured by a tensiometer (DCAT 25, DataPhysics, Germany). In addition to water droplets containing PEO,^19−21,23−25^ we also tested various complex liquids made with aqueous solutions of 6 wt % polyacrylamide (PAAM, Macklin, China)^22,26^ and 2 wt % konjac glucomannan (KGM, Aladdin, China)^38^ to demonstrate the wide applicability of 3S surfaces.

## Results and Discussion

### Demonstration of Surface Superhydrophobicity by Passive Water Repellency

We start the demonstration of the superhydrophobicity–slipperiness switchable surface (3S surface) with its superhydrophobicity, that is, the capability of repelling water droplets. In this section, we will show the removal of water droplets by the spontaneous motion of droplets (passive repellency) and by the active motion of surface structures (active repellency).

**Figure 4:**
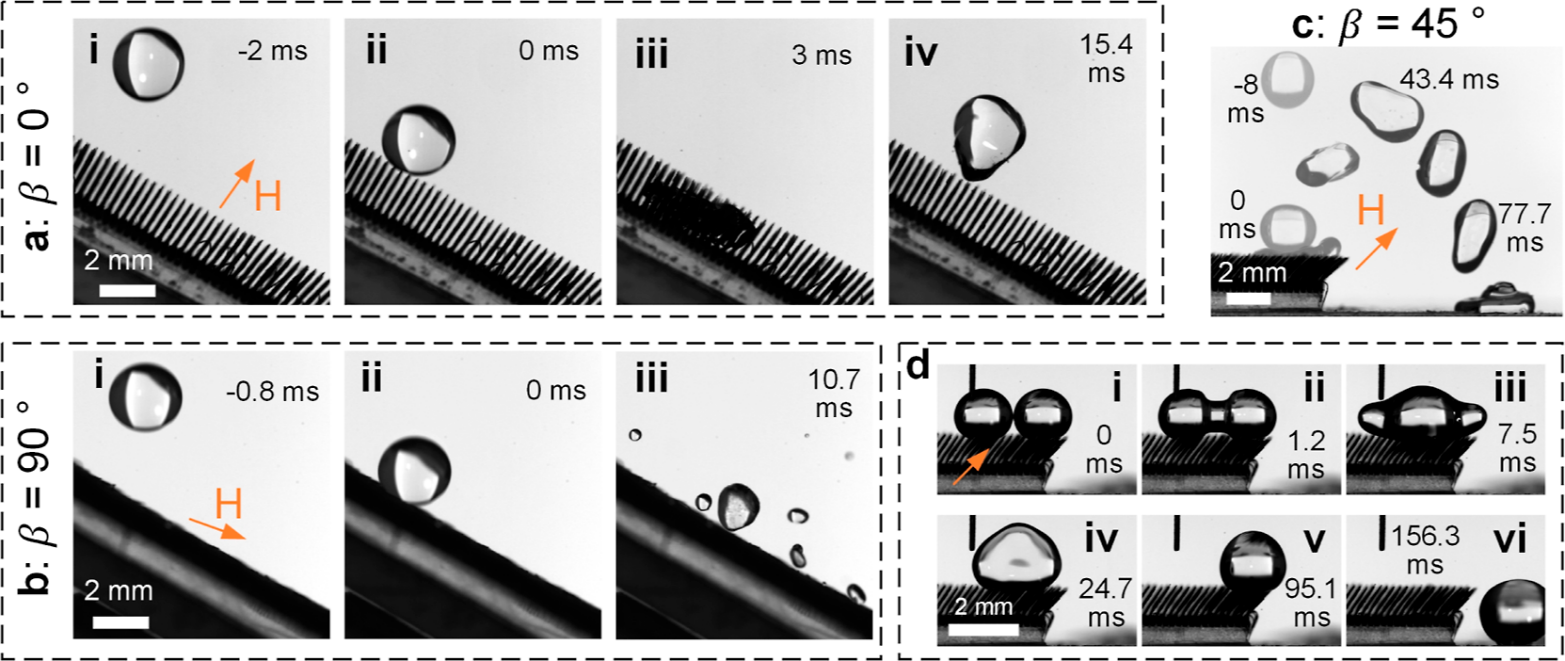
Demonstration of 3S surface’s superhydrophobicity by droplet impact. (a) Impact dynamics of a water droplet released at 10 cm onto the 3S surface with vertical wires (β ≈ 0°) and (b) those of a water droplet released at 70 cm onto the 3S surface with fully inclined wires (β ≈ 90°), where both surfaces are tilted at 30°. (c) Directional bouncing of an impacting water droplet released at 10 cm and (d) coalescence-induced droplet removal from the 3S surface with wires inclined at β ≈ 45°, where the 3S surface is horizontally placed. The moment at the initial droplet–substrate and droplet–droplet contact is denoted as *t* = 0 ms. The direction of the magnetic field lines (*H*) is indicated by the arrows.

To demonstrate passive water repellency, the impact dynamics of water droplets at room temperature are shown in Figure 4a. When the magnetic field was perpendicularly applied to a 30°-titled 3S surface (the wires are perpendicular to the surface with wire inclination angle β ≈ 0°), the 10 μL droplet released at a height of 10 cm (corresponding impact speed at 1.4 m/s) fully wetted the surface structures in the millimetric scale (including blades and wires) and rebounded with a droplet–substrate contact time of 15.4 ms.^39^ The droplet cannot wet the surface structures in the microscopic scale (SiO_2_ nanoparticles) because the dynamic pressure of the impacting droplet cannot overcome the capillary pressure resisting liquid penetration, a term inversely proportional to the size of voids between structures,^40^ provided by the nanoparticles. Therefore, the droplet can still rebound, as the droplet–surface adhesion is still weak. Nevertheless, droplets with higher impact speeds (e.g., impacting speed at 1.95 m/s and release height of 20 cm) failed to rebound from the 3S surface and remained pinned with this wire configuration (Figure S4). We speculate that the deformation and vibration of flexible wires led to a large energy dissipation that prevented droplet rebound.^41,42^

Therefore, another surface configuration is tested, where the wires are completely inclined (β ≈ 90°) by relocating the magnetic field. Since the inclined wires stacked on each other fully blocked the voids between them [Figure 3b(ii),c(ii)], the impacting water droplet hardly penetrated the wire matrix, and hence the droplet mainly interacted with the SiO_2_ nanoparticles on the wire sidewalls. In other words, the 3S surface with wires of β ≈ 90° can be considered as a flat superhydrophobic surface in the millimetric length scale. As a result, the droplet fully rebounded and splashed from the 3S surface without any residue left, even though the release height was increased to 70 cm with a corresponding impact speed of 3.7 m/s (Figure 4b). The contact time of 10.7 ms in Figure 4b is much smaller than that of 15.4 ms in Figure 4a because droplet retraction did not occur due to splashing.

The above successful demonstrations of water repellency were performed with tilted 3S surfaces, which may not be applicable to applications that do not permit surface inclination. For example, an impacting droplet may repeat impact–rebound cycles but eventually land on the horizontally placed surface. To address this issue, the water droplet was allowed to impact a horizontally placed 3S surface with partially inclined wires (β ≈ 45°). As shown in Figure 4c, the impacting droplet rebounded with a horizontal displacement in line with the wire inclination, a phenomenon known as the directional bouncing.^15^ This is because the liquid in between wires retracted following the wire inclination, leading to a directional momentum that drove the droplet.^43,44^ Moreover, as shown in Figure 4d, the coalescence of two droplets released a substantial surface energy, which got transferred to the kinetic energy, resulting in droplet vibration.^45^ The droplet vibration was accompanied by the periodic advancing and retraction of the contact line, which experienced smaller and larger resistance in the motion direction along and against the wire inclination, respectively.^17,46,47^ This anisotropic contact line dynamics (the break of symmetry) led to the droplet directional motion and more detailed elaborations can be found in prior studies.^9,48−51^

The demonstrations in Figure 4 reveal that water droplets can be passively repelled by our 3S surface thanks to the low droplet–substrate adhesion and the magneto-responsiveness of the wire that allows a significant decrease in droplet–substrate contact area in response to a magnetic field. In addition to such a passive water repellency, we will manifest the surface superhydrophobicity by showing the removal of impurities in an active manner.

### Demonstration of Surface Superhydrophobicity by Active Impurity Removal

**Figure 5:**
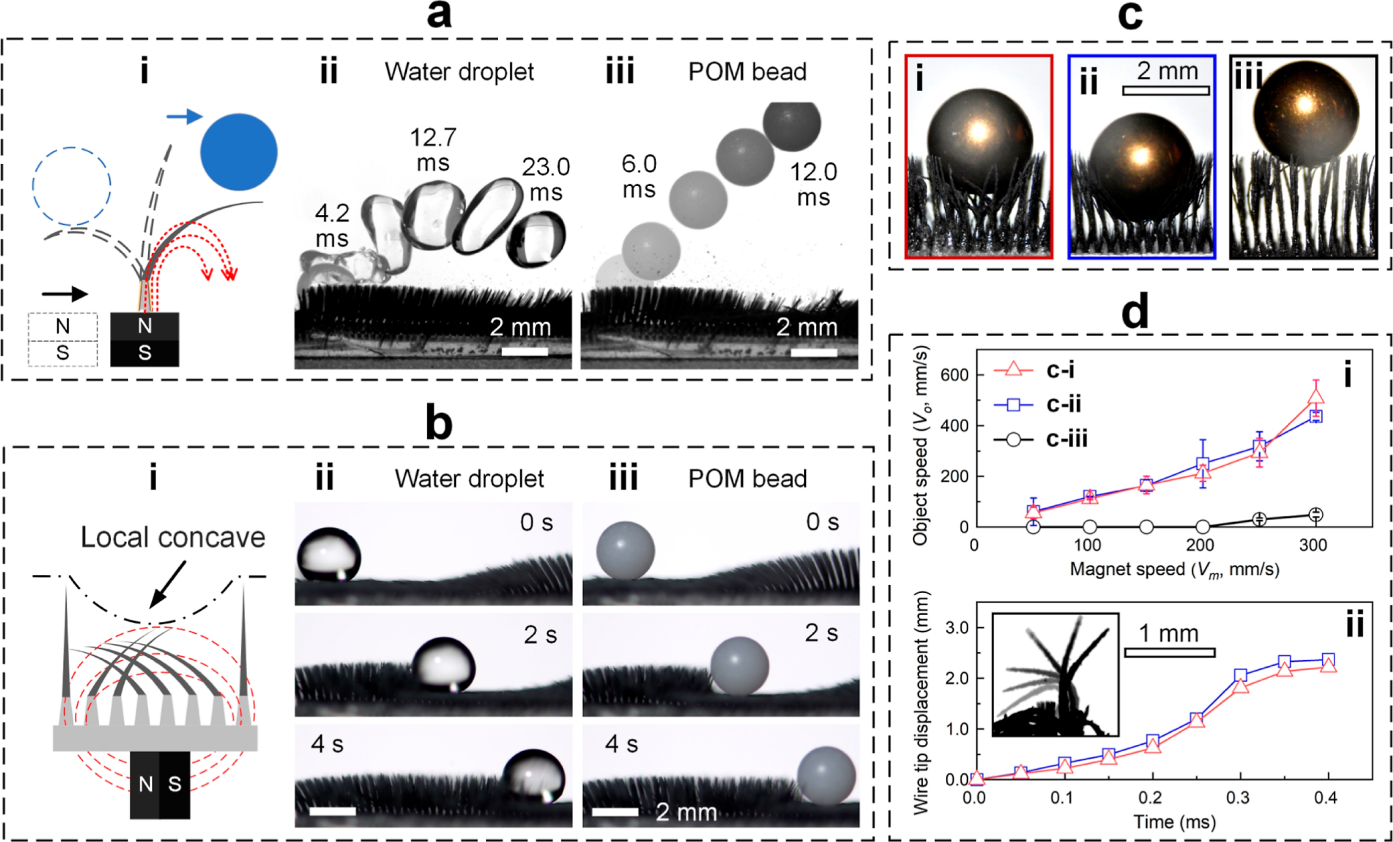
Demonstration of 3S surface’s superhydrophobicity by active removal of impurities. (a) Fast impurity removal by a moving magnet at a speed of 300 mm/s: (i) schematics of the mechanism, removal of a (ii) water droplet and (iii) POM bead. (b) Precise impurity removal by a moving magnet at a speed of 2 mm/s: (i) schematics of the mechanism, manipulation of a (ii) water droplet and (iii) POM bead. (c) Wire configurations when placing a 68 mg copper bead atop 3S surfaces with different wire stiffnesses: (i) regular wires prepared using the as-reported methods in this study, (ii) wires without the coating of nanoparticles or impregnation of paraffin, and (iii) wires with additional coating of epoxy. (d) Measured (i) speeds of the removed object with respect to the magnet speeds and (ii) wire tip displacement of wires shown in [c(i,ii)] with time caused by a magnet sliding at 300 mm/s. The inserted image in [d(ii)] is a time-lapse image of the motion of a wire.

The magneto-responsive wires can quickly move in response to a fast-changing magnetic field. As shown in Figure 5a, a 10 μL water droplet and a 2.5 mm-diameter POM bead (both are considered as impurities) placed on a horizontal 3S surface were removed when a magnet, whose north and south poles were placed perpendicular to the 3S surface, slid underneath the 3S surface at 300 mm/s. This is because the fast-changing magnetic field caused by the moving magnet led to fast-swinging wires, which transferred the kinetic energy from the wires to that of the impurity by impingement [Figure 5a(i)]. The phenomenon of impurity removal is more significant for POM beads [Figure 5a(iii)] than that for water droplets [Figure 5a(ii)] because the liquid–solid adhesion (a capillary effect) must be overcome.^17^

Different from the above mechanism, which involves the transfer of kinetic energy from the swinging wires to the impurities, impurities (objects) can be removed (manipulated) in a more controlled manner. Specifically, the north and south poles of the magnet are placed in parallel to the 3S surface, and the magnetic field lines in this configuration cause a local concave of the wire arrays on which the object tends to stay under the effects of gravity [Figure 5b(i)]. As such, the impurities moved to the valley, whose motion was identical to the magnet and no difference in speeds between water droplets and solid objects can be found [Figure 5b(ii,iii)].^17^

The difference between those two modes of impurity removal is determined by the configuration of the magnetic field lines, which can be changed by adjusting the alignment of a magnet’s north and south poles with respect to the 3S surface. Moreover, our prior work suggested that the two modes have overlapped operation conditions from 20 to 100 mm/s, below which the first mode failed because the kinetic energy of the wires was insufficient to mobilize the impurity, and above which the latter mode failed because the object cannot catch up with the motion of the local concave.^17^

We also examined the effect of wire stiffness on the active removal of impurities. Although it is difficult to precisely control and measure the stiffness or Young’s modulus of a single wire, compared with the 3S surface prepared using the as-reported method [Figure 5c(i)], we decreased the wire stiffness by skipping the coating of nanoparticles and the impregnation of paraffin [Figure 5c(ii)] and increased the wire stiffness by adding 6 wt % epoxy (epoxide resin and its curing agent) in the solution of nanoparticle coatings [Figure 5c(iii)]. The variation of wire stiffnesses was reflected by placing a 68 mg of copper bead atop those 3S surfaces without a magnetic field applied (Figure 5c). For wires that are sufficiently soft [Figure 5c(i,ii)], the wires demonstrated identical performance for fast object removal regardless of the variations in wire stiffness [Figure 5d(i)] because the wire dynamics, e.g., trajectories of wire tips [Figure 5d(ii)], are fully dictated by the magnetic fields. For wires exceeding a critical stiffness [Figure 5c(iii)], whose specific value is out of the scope of this study, the moving magnetic field barely mobilized the wires, and hence the impurity removal can be noticed only when the magnet reached a moving speed of as large as 250 mm/s [black symbols in Figure 5d(i)].

### Failure of Surface Superhydrophobicity in Repelling Complex Droplets

**Figure 6:**
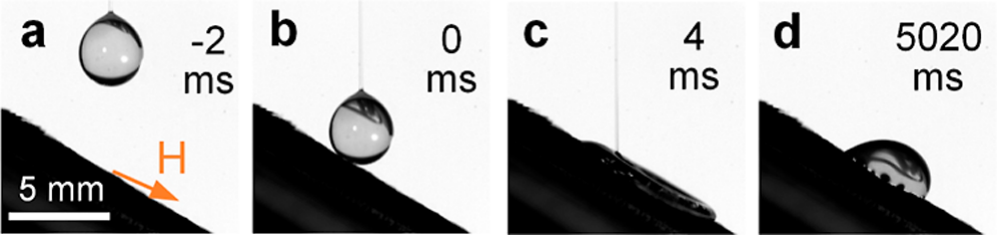
Failure of the 3S surface in repelling a water droplet containing 2 wt % PEO: the (a) droplet before touching the substrate, (b) droplet at the initial droplet–substrate contact, (c) droplet at the maximum spreading, and (d) static droplet remained anchored on the substrate. The droplet volume is 20 μL and the impact speed is 2.1 m/s.

However, the above functionalities of the 3S surface fail when the object of interest is a complex droplet (a water-based droplet containing macromolecules, that is, a diluted polymer solution) rather than a pure water droplet. Specifically, as shown in Figure 6, a water droplet (∼20 μL) containing 2 wt % PEO at an impacting speed of 2.1 m/s failed to rebound and remained pinned on the 30°-tilted 3S surface with completely deflected wires β ≈ 90° (see also Video S1 in Supporting Information). This behavior shows a dramatic contrast to what is observed in Figure 4b, where the pure water droplet with an impacting speed as high as 3.7 m/s completely bounced-off from the 3S surface. Since the completely inclined wires β ≈ 90° and relatively large liquid–gas interfacial tension (γ_LG_ of ∼0.063 N/m) ensured that the complex droplet contacted only the wires rather than wetting the sidewalls or the bottoms of the blades, the Wenzel wetting state in the millimetric scale or the wire vibration should be excluded from the reasons that led to the pinning of complex droplets. In other words, the Cassie–Baxter wetting state was still sustained for the PEO-laden droplet in Figure 6. Therefore, the pinning of complex droplets should be caused by reasons relating to macromolecules in the droplet, e.g., rheological characteristics within the bulk droplet and near the contact line and macromolecule–substrate interactions,^19−26^ which cannot be resolved by merely enhancing surface superhydrophobicity or its robustness.

The experimental results suggest that various surfaces with excellent water repellency (Figures 2a,b and 4b) all failed in repelling droplets containing macromolecules (Figures 2c,d and 6), highlighting the severity of this problem. As such, in the next section, we will introduce our new approach that resolves this problem.

### Demonstration of Surface Superhydrophobicity-To-Slipperiness Transition

**Figure 7:**
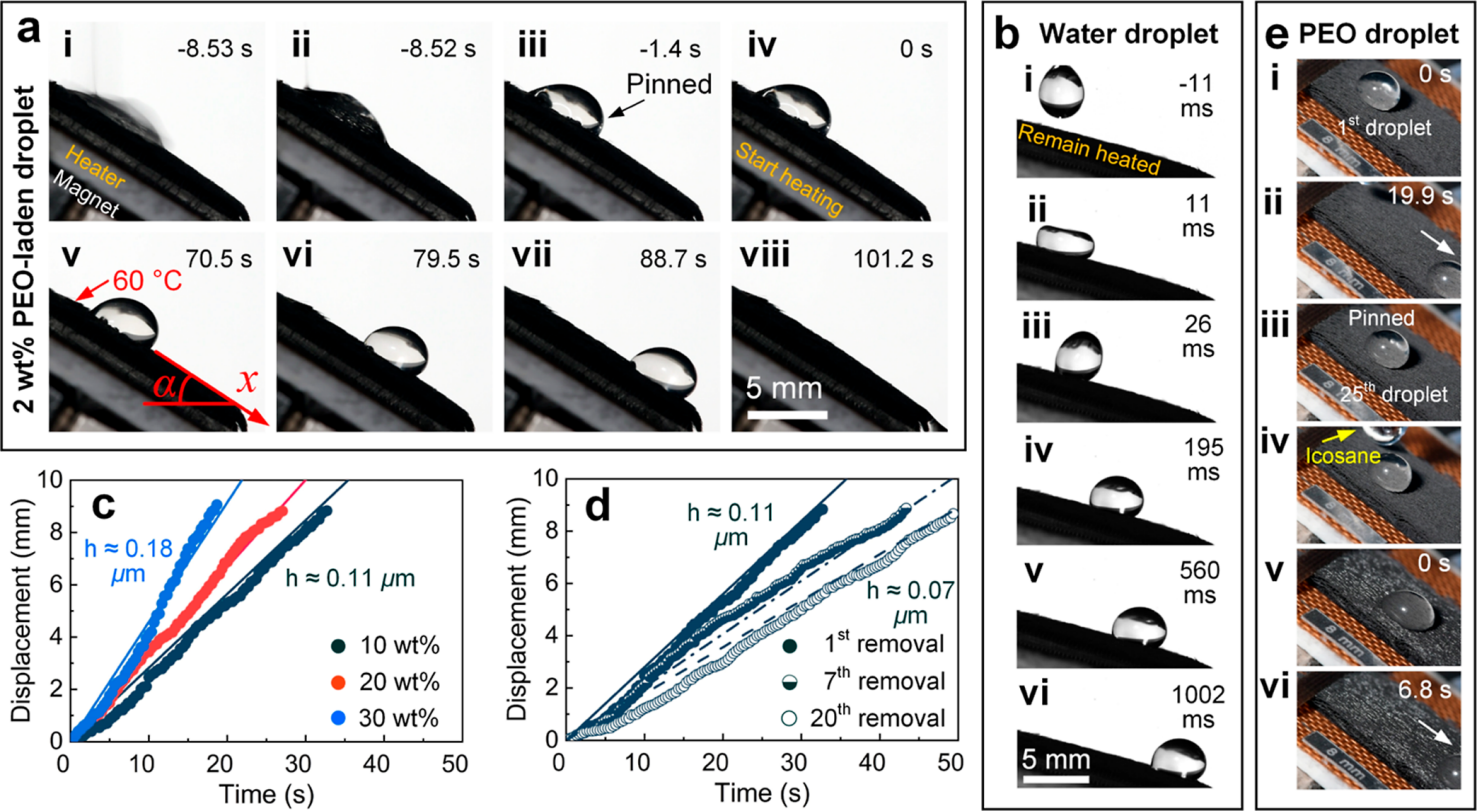
Demonstration of the superhydrophobicity-to-slipperiness transition of the 3S surface for the removal of complex droplets and the characterization of surface slipperiness. (a) Slide-off of the previously pinned PEO-laden droplet from the 3S surface when the surface substrate is heated at 60 °C, where the onset of heating is denoted as *t* = 0 s. (b) Sliding of a water droplet on a heated 3S surface tilted at 15°. (c) Displacement of PEO-laden droplets on 3S surfaces impregnated with different amounts of paraffin. (d) Displacement of PEO-laden droplets on a 3S surface impregnated with 10 wt % paraffin at different superhydrophobicity-to-slipperiness switch cycles. The solid and dashed lines are the fitting lines using eq 3 to estimate the paraffin layer thickness *h*. (e) Loss of mobility of the 25th PEO-laden droplet on the heated (60 °C) 3S surface and the resumption of its mobility by replenishing paraffin.

For the same case shown in Figure 6, where a 2 wt % PEO-laden droplet with a volume of ∼20 μL remained pinned on the 3S surface with inclined wires β ≈ 90°, the surface was heated by an electrothermal heater from its bottom. As shown in Figure 7a(iv), where *t* = 0 s is defined as the onset of heating, after a period from 30 to 60 s, we observed that the pinned droplet recoiled from its flattened shape with its diameter decreasing and the contact angle at its uphill side increasing [Figure 7a(v)]. The droplet recoil indicated the existence of lubricants on the surface (paraffin was melted on the wires), which eased contact line pinning. This period (from 30 to 60 s) represents the time probably required to warm up the electrothermal heater, transferring the heat from the heater to the bottom of the 3S surface and then to the wires, and the melting of sufficient paraffin.

Then, after few seconds, the sliding of the PEO-laden droplet was observed (from Figure 7a(v–viii)), indicating that a substantial amount of melted paraffin appeared in between the 3S surface and the PEO-laden droplet by forming a lubricant layer (see also Video S1). To corroborate the existence of this lubricant layer and characterize the surface slipperiness, 20 μL water droplets were continuously released from a height of 2 mm and at a rate of six droplets per second to impact a heated, 15°-tilted 3S surface. As shown in Figure 7b and Video S2, water droplets cannot rebound (vertically detach) from the surface but continuously slid off, indicating a successful transition from the superhydrophobic surface to the lubricant-impregnated surface by thermoinduced melting of the impregnated paraffin. In fact, we have also tested various complex droplets which remained pinned on conventional superhydrophobic surfaces, such as PAAM-laden^22,26^ and KGM-laden^38^ droplets, and the successful removal of them are demonstrated in Figure S5.

### Characterization of Surface Slipperiness

We then analyze the effects of impregnated paraffin on the dynamics of droplet removal, that is, droplet displacement with respect to time. Figure 7c shows the droplet displacement on 3S surfaces impregnated with various amounts of paraffin (∼10, ∼20, and ∼30 wt %). We observed that after the PEO-laden droplet started to slide (defined as *t* = 0 s), the PEO-laden droplet moved faster on the 3S surface impregnated with more paraffin (∼30 wt %) than the case with less impregnated paraffin (∼10 wt %). By ignoring factors such as dissipations within the droplet and along the droplet–paraffin–gas contact line, this relationship can be qualitatively explained by the gravitational force acting on the droplet and the viscous friction force of the lubricant layer, which depends on a constant thickness of the paraffin layer *h* in between the droplet and the inclined wires, as
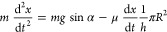
1where *m* is droplet mass, α is the tilt angle of the substrate, μ is the viscosity of the lubricant, π*R*^2^ is the measured droplet base area on which the frictional force acts, and d*x*/(d*t·h*) is the shear rate. Using the boundary conditions of *x*(0) = 0 and *x*′(0) = 0, the droplet displacement *x* can be expressed as
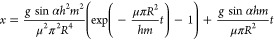
2By neglecting the higher-order term (*hm*/μπ*R*^2^)^2^, that is, the first term on the right-hand side, the droplet speed linearly depends on the paraffin layer thickness *h* as
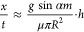
3Fitting eq 3 to the measured droplet displacement *x* against time *t* shown in Figure 7c, a clear increase of the fitted *h* with the increase in the amount of impregnated paraffin can be observed. Specifically, the surface impregnated with ∼10, ∼20, and ∼30 wt % paraffin corresponds to the fitted *h* of 0.11, 0.13, and 0.18 μm, respectively. More impregnated paraffin led to a larger film thickness *h*, a smaller shear rate d*x*/(d*t·h*), a smaller viscous friction force, and therefore a larger droplet speed.

We then explore the repeatability of the 3S surface for superhydrophobicity-to-slipperiness transition, namely, the effects of removal cycles on droplet removal dynamics. A cycle includes the impingement of a complex droplet onto the 3S surface, the droplet pinning, substrate heating and paraffin melting, droplet removal, the substrate cooling, and paraffin solidification with the demonstration of surface superhydrophobicity. Using the 3S surface with 10 wt % paraffin as an example, Figure 7d shows a decreasing droplet sliding speed with an increasing repeated cycle. This can also be explained by eq 3, where the decrease in speed is attributed to the decrease in paraffin layer thickness *h* as the paraffin has been depleted by sliding droplets. We then fitted the measured droplet displacement *x* over time *t* using eq 3 to get the film thickness *h*. A decrease of *h* with an increase in repeated cycles can indeed be observed, where the 1st, 7th, and 20th droplet removals correspond to the fitted *h* of 0.11, 0.08, and 0.07 μm, respectively. Nevertheless, it should be noted that eq 1 is simplified to show the qualitative effects of the amount of impregnated paraffin on the removal of complex droplets, and hence the measurement or investigation of the actual paraffin film thickness is out of the scope of this study.

We then allowed continuous sliding of 20 μL PEO-laden droplets every 20 s on a heated 3S surface impregnated with 10 wt % paraffin, as shown in Figure 7e. Since the melted paraffin layer can be depleted by sliding complex droplets, it is expected to see the pinning of the 25th PEO-laden droplet [Figure 7e(iii)]. Nevertheless, the impregnated paraffin can be replenished in situ. As shown in Figure 7e(iv), around 50 μL of melted icosane (paraffin) was added onto the uphill side of the pinned droplet, and then the pinned droplet quickly resumed its mobility, and the subsequent removals of more than 100 droplets can be observed because the newly added icosane remained mainly on the surface top.

Lastly, we discuss the limitations and future investigations of the developed 3S surface. Regarding durability, the magneto-responsive wires cannot withstand mechanical scratches or rubbing because they are made with PDMS. Nevertheless, by swinging the wires for 0.1 million cycles using a magnet that traveled back and forth, no deterioration in functionality can be noticed.^17^ The optimization between flexibility and mechanical durability should be conducted in future studies. Moreover, iron (Fe) particles within wires can be removed by acid solutions (e.g., HCl), and the superhydrophobic coatings of SiO_2_ nanoparticles can be damaged by alkali solutions (e.g., NaOH), causing the loss of magneto-responsiveness and superhydrophobicity, respectively.^17^ Regarding the operating conditions, the 3S surface reported in this study was impregnated with icosane (C_20_H_42_) with the melting temperature at ∼36 °C, and therefore the superhydrophobicity–slipperiness switch cannot be allowed under conditions with temperature over paraffin’s melting point. Nevertheless, there are various types of paraffin with different melting points, and hence the 3S surface can be customized for various operating conditions.^34^ Regarding scalability, the 3S surface for demonstration purposes has a size of a few centimeters, which can be scaled up by using a magnetic field with a larger size and an atomizer that produces a larger spray of iron-laden aerosols. This is because the magneto-responsive wires are formed from the self-assembly of iron-laden aerosols under a magnetic field.^17^

## Conclusions

Superhydrophobic surfaces with excellent functionalities such as passive water repellency and active impurity removal have attracted extensive attention because of their great potential in interface-related applications. However, it is a long-standing problem that superhydrophobic surfaces have difficulties in repelling water droplets containing macromolecules (complex liquids),^19−21^ which are pervasive in practical applications ranging from our daily lives, biomedical devices, chemical plants, to petroleum industry. Pitcher plant-inspired lubricant-impregnated surfaces (slippery surfaces) allow sliding off of many complex liquids, but a surface inclination is typically required. A successful integration of surface slipperiness and superhydrophobicity has rarely been reported because the former requires a lubricant layer atop the solid structures (a liquid–lubricant–gas system) and the latter requires a minimized solid–liquid contact with air pockets trapped in between (a liquid–solid–gas composite system)—they are inherently contradictory with each other.

To combat this problem, here, we designed a surface termed superhydrophobicity–slipperiness switchable surface (3S surface), which was made with discrete, well-ordered, high-aspect-ratio, and iron-laden wires and was also coated with hydrophobic nanoparticles and impregnated with paraffin, exhibiting superhydrophobicity at room temperature and slipperiness when heated. At room temperature, the 3S surface with solidified paraffin (a liquid–solid–gas composite system) demonstrated excellent superhydrophobicity by allowing water droplet rebound/splashing at high impacting speeds (the tested impacting speeds up to 3.7 m/s), directional droplet bouncing upon impact, and directional droplet removal upon coalescence—excellent passive repellency of water. Moreover, powered by a moving magnetic field, surface impurities can be removed by magneto-responsive wires—excellent active repellency of impurity. When encountering various complex droplets that remained pinned on superhydrophobic surfaces,^19−26,38^ the 3S surface was heated, and the melted paraffin formed a lubricant layer in between the inclined wires and the complex droplets (a liquid–lubricant–gas system). This led to a continuous removal of various complex droplets under gravity—surface slipperiness. We believe the designed magneto- and thermo-responsive structures, which counterintuitively manifest the advantageous characteristics of both superhydrophobic surfaces and lubricant-impregnated surfaces and the switch between them, will have great potential in various interface-related applications.

## Supporting Information Available

Schematics of the fabrication process of the 3S surface; dependence of the weight percentages of the impregnated paraffin on the paraffin-removing time; viscoelastic properties of the complex liquids; the failure of droplet rebound and removal from the 3S surface with wires perpendicular to the substate; and the pinning and removal of various complex droplets on the 3S surface (PDF)
Pinning and removal of a PEO-laden droplet on a 3S surface (MP4)
Continuous removal of water droplets from a heated 3S surface (MP4)
