# Supplementary material for: A Superhydrophobicity–Slipperiness Switchable Surface with Magneto- and Thermo-responsive Wires for Repelling Complex Droplets
Source: Langmuir. 2024 Jan 22;40(5):2764–72. doi: 10.1021/acs.langmuir.3c03556 (PMC10851661; doi:10.1021/acs.langmuir.3c03556)
Supplement: Supplementary file 1 — la3c03556_si_001.pdf [file la3c03556_si_001.pdf]

Supporting Information

A Superhydrophobicity-Slipperiness Switchable Surface with Magneto- and Thermo-Responsive Wires for Repelling Complex Droplets

Chuanqi Wei[1], *Oleg Gendelman*[2*], *Youhua Jiang*[1,2,3*]

1. Department of Mechanical Engineering (Robotics), Guangdong Technion – Israel Institute of Technology, Shantou, Guangdong 515063, China
2. Faculty of Mechanical Engineering, Technion – Israel Institute of Technology, Haifa 3200003, Israel
3. Guangdong Provincial Key Laboratory of Materials and Technologies for Energy Conversion, Guangdong Technion – Israel Institute of Technology, Shantou, Guangdong 515063, China

Fig. S1. Schematics of the fabrication process of the 3S surface. 2

Fig. S2. Dependence of the weight percentage of the impregnated paraffin on the paraffin-removing time. 3

Fig. S3. Rheological properties of the complex liquids.. 4

Fig. S4. The failure of droplet rebound and removal from the 3S surface with wires perpendicular to the substrate. 5

Fig. S5. The pinning and removal of various complex droplets on the 3S surface. 6

[*] To whom correspondence should be addressed. E-mail: youhua.jiang@gtiit.edu.cn and ovgend@me.technion.ac.il

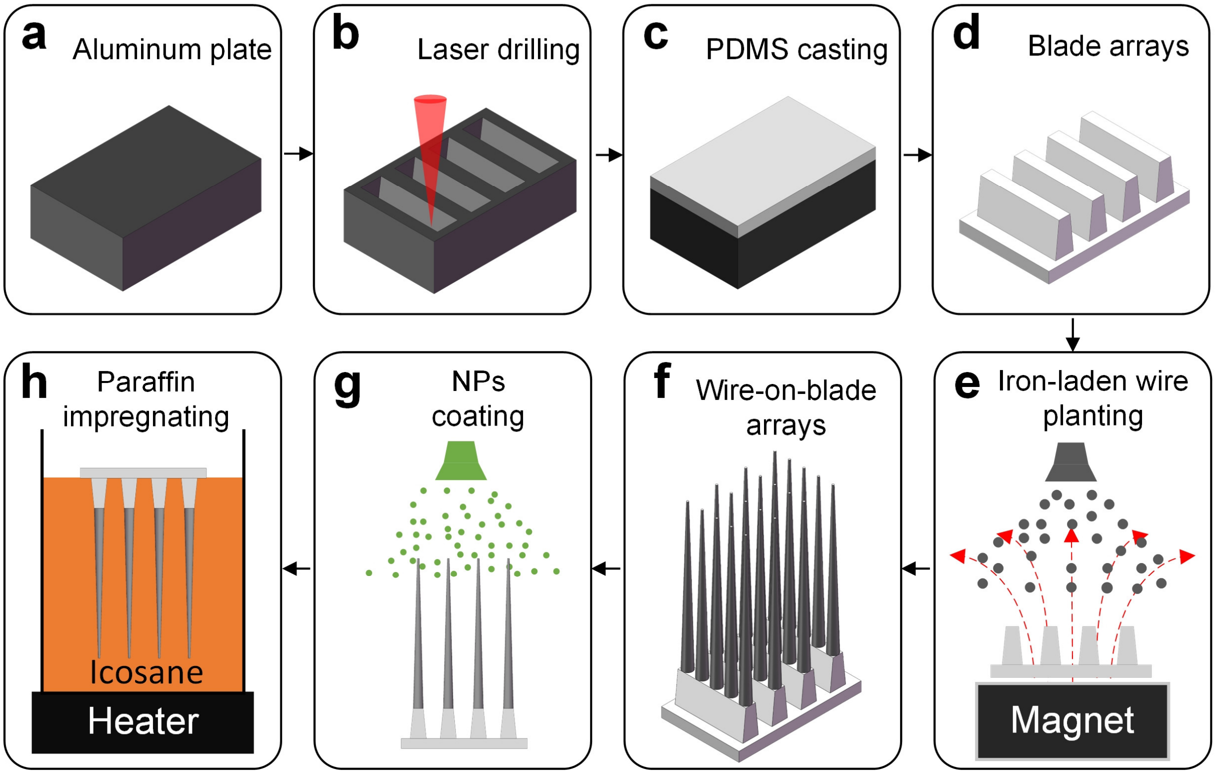

Fig. S1. Schematics of the fabrication process of 3S surface: (**a** and **b**) production of grooves on an aluminum plate by laser drilling; (**c** and **d**) replication for PDMS blade arrays; (**e** and **f**) formation of magneto-responsive iron-laden PDMS wires atop blade arrays by self-alignment of iron-laden aerosols under a magnetic field (0.45 T) followed by heat-curing; (**g**) surface super-hydrophobization by spray-coating hydrophobic SiO_2 (7-40 nm) nanoparticles; (**h**) impregnation of paraffin (icosane) into surface structures by immersing the surface into a paraffin liquid heated at 80 °C for 1 hour.

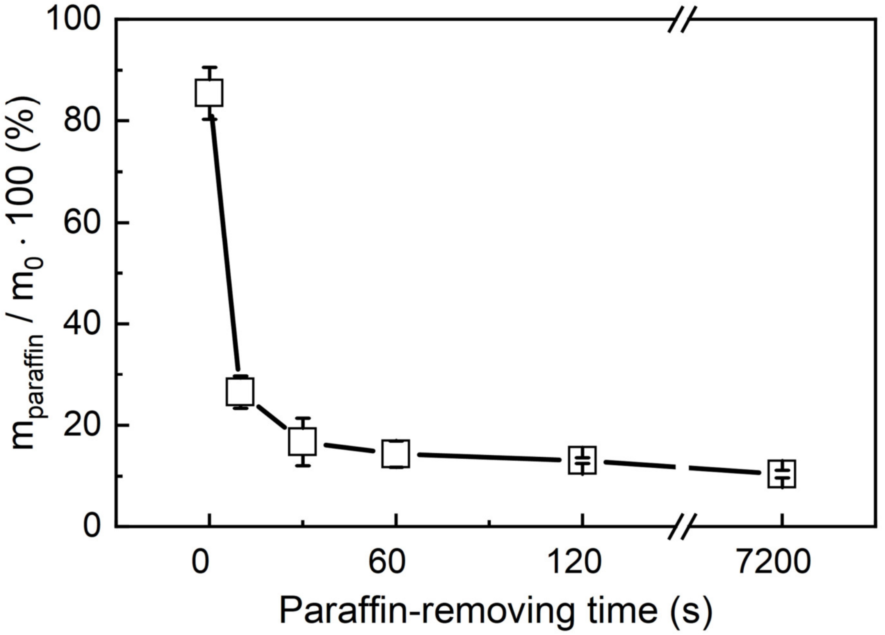

Fig. S2. The weight percentages of the impregnated paraffin as compared to the surface weight after paraffin-impregnation are plotted with respect to the paraffin-removing time. $m_{paraffin}$ is the weight of impregnated paraffin and m_0 is the weight of processed surface (the surface after impregnating into the liquid paraffin tank and after paraffin-removing).

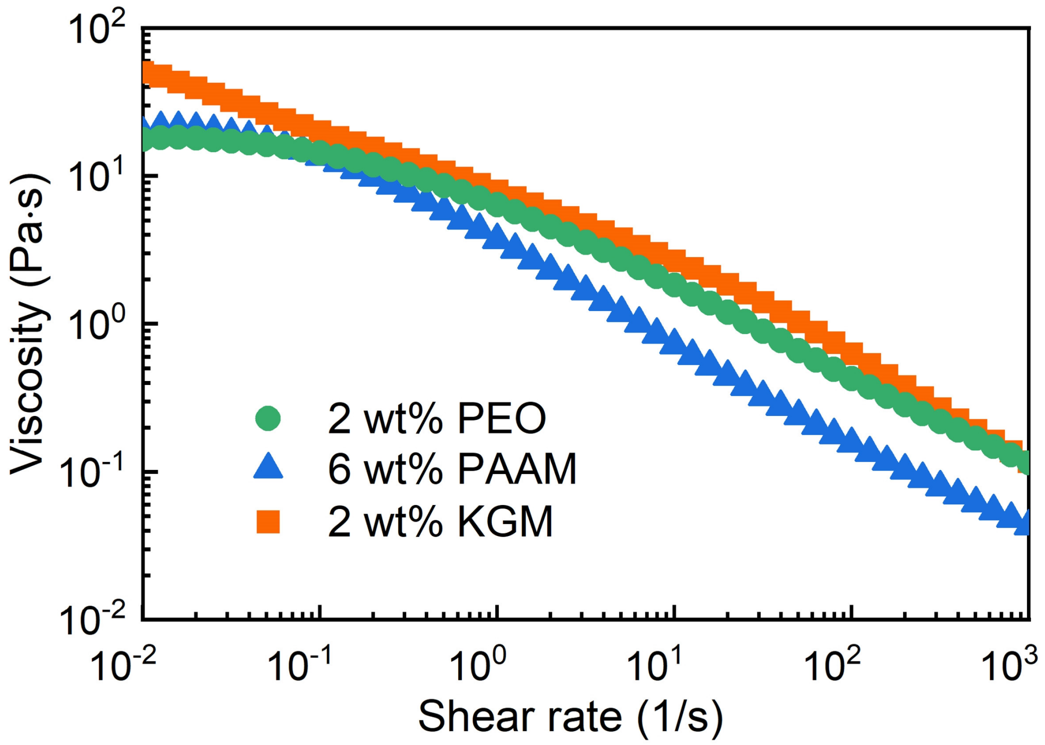

Fig. S3. Measured viscosity of the complex liquids, e.g., 2 wt% PEO, 6 wt% PAAM, and 2 wt% KGM, with respect to the shear rate.

The molecular weight of poly(ethylene oxide) (PEO) and polyacrylamide (PAAM) is $4 \cdot 10^6$ g/mol and $5 \cdot 10^6$ g/mol, respectively. The molecular weight of konjac glucomannan (KGM) ranges from $2 \cdot 10^5$ to $2 \cdot 10^7$ g/mol. The liquid-gas interfacial tensions γ_{LG} of 2 wt% PEO, 6 wt% PAAM, and 2 wt% KGM were measured to be 0.063 N/m, 0.039 N/m, and 0.056 N/m, respectively. Shear-dependent viscosity of 2 wt% PEO, 6 wt% PAAM, and 2 wt% KGM ranges from 17.64 Pa·s to 0.12 Pa·s, 19.56 Pa·s to 0.04 Pa·s, and 50.17 Pa·s to 0.12 Pa·s, respectively, for the shear rate ranging from 0.01 1/s to 1,000 1/s.

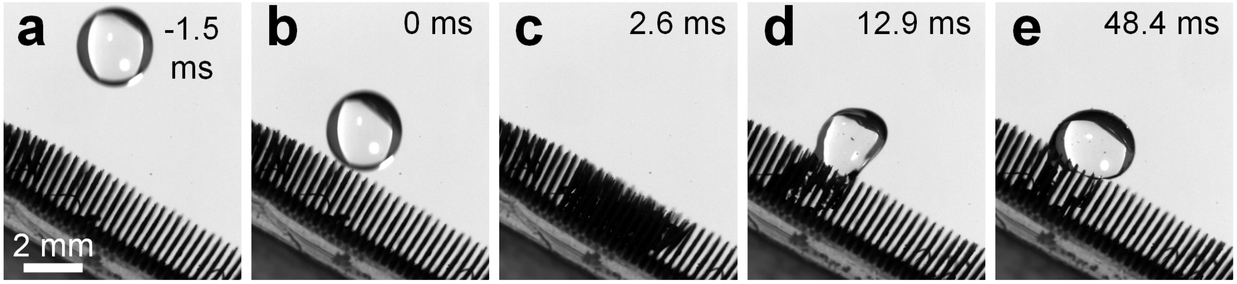

Fig. S4. When a water droplet is released at 20 cm, the failure of droplet rebound and removal from a 30°-tilted 3S surface with wires perpendicular to the substrate ($\beta \approx 0°$): (**a**) the droplet before touching the substrate, (**b**) the droplet at the initial droplet-substrate contact, (**c**) the droplet at the maximum spreading, (**d**) the droplet that tended to rebound from the substrate, and (**e**) the droplet that failed to rebound from the substrate.

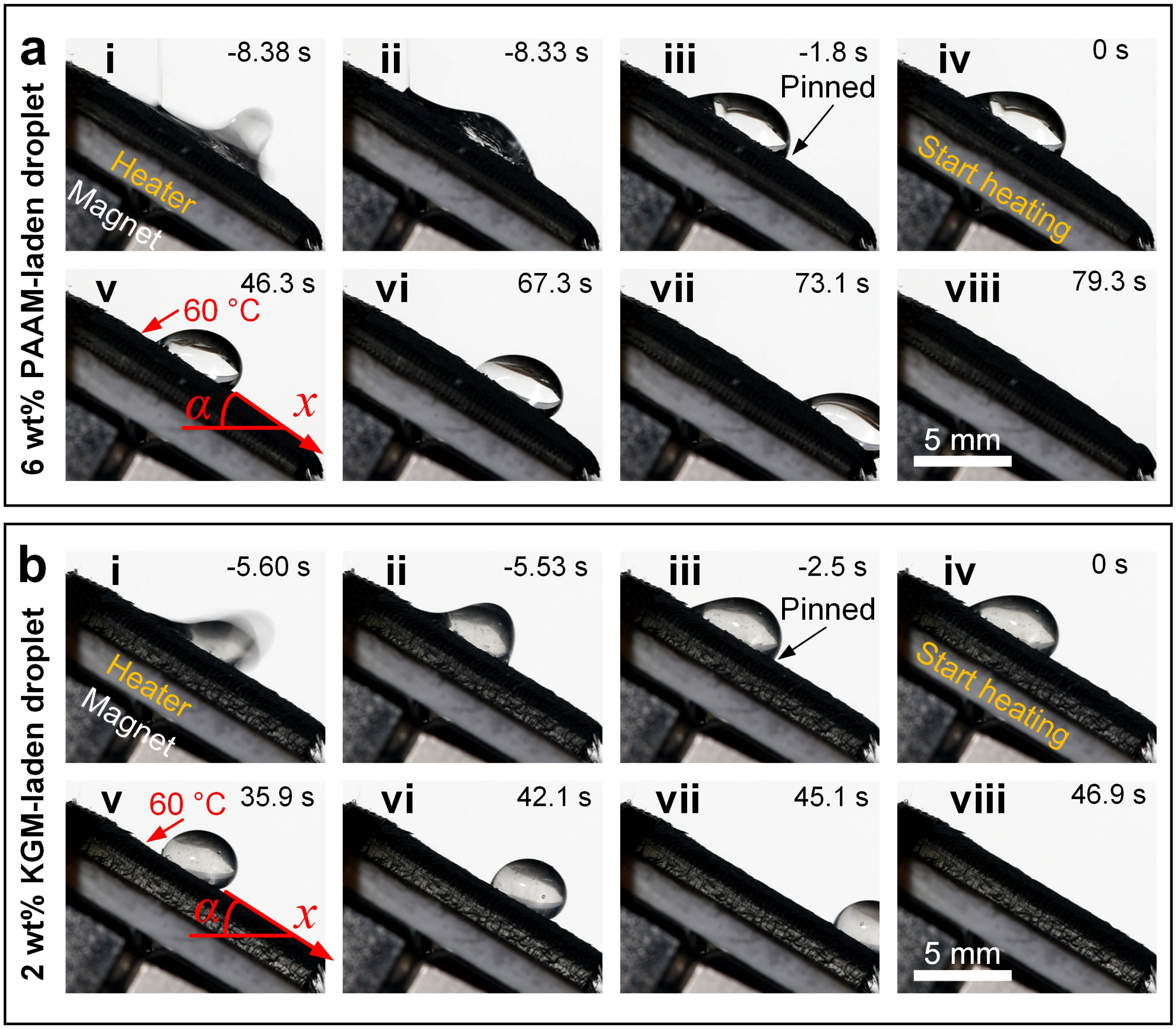

Fig. S5. The failure of the 3S surface in repelling water droplets containing various macromolecules at room temperature: (from **a-i** to **a-iii**) 6 wt% PAAM and (from **b-i** to **b-iii**) 2 wt% KGM. The slide-off of the pinned droplets by superhydrophobicity-to-slipperiness transition: (from **a-iv** to **a-viii**) 6 wt% PAAM and (from **b-iv** to **b-viii**) 2 wt% KGM. Droplet volumes and impacting speeds in (**a**) are 22 μL and 2.3 m/s and those in (**b**) are 22 μL and 2.8 m/s, respectively.
